# Electrical and optical properties of epitaxial binary and ternary GeTe-Sb_2_Te_3_ alloys

**DOI:** 10.1038/s41598-018-23221-9

**Published:** 2018-04-12

**Authors:** Jos E. Boschker, Xiang Lü, Valeria Bragaglia, Ruining Wang, Holger T. Grahn, Raffaella Calarco

**Affiliations:** 1 Paul-Drude-Institut für Festkörperelektronik, Leibniz-Institut im Forschungsverbund Berlin e. V., Hausvogteiplatz 5−7, 10117 Berlin, Germany; 2 Leibniz Institute for Crystal Growth, Max Born Str. 2, 12489 Berlin, Germany

## Abstract

Phase change materials such as pseudobinary GeTe-Sb_2_Te_3_ (GST) alloys are an essential part of existing and emerging technologies. Here, we investigate the electrical and optical properties of epitaxial phase change materials: α-GeTe, Ge_2_Sb_2_Te5 (GST225), and Sb_2_Te_3_. Temperature-dependent Hall measurements reveal a reduction of the hole concentration with increasing temperature in Sb_2_Te_3_ that is attributed to lattice expansion, resulting in a non-linear increase of the resistivity that is also observed in GST225. Fourier transform infrared spectroscopy at room temperature demonstrates the presence of electronic states within the energy gap for α-GeTe and GST225. We conclude that these electronic states are due to vacancy clusters inside these two materials. The obtained results shed new light on the fundamental properties of phase change materials such as the high dielectric constant and persistent photoconductivity and have the potential to be included in device simulations.

## Introduction

Phase change materials (PCMs) such as GeTe-Sb_2_Te_3_ (GST) alloys^1^ are extensively used and investigated for their application as a storage medium in optical discs and in phase change random access memory^2,3^. These two applications represent mature technologies that rely on the optical and electrical contrast between the amorphous and crystalline phases of PCMs and the ability to switch fast, reversibly, and reliably between them^4^. In recent years, novel applications have emerged that also rely on the property contrast offered by PCMs. For example, it has been demonstrated that PCMs can be used to fabricate high-resolution optical displays^5^. Another interesting new application is the use of PCMs as on-chip, non-volatile photonic memories^6^. Furthermore, simulations have shown that metadevices based on PCMs can be used as modulators and absorbers in the near-infrared (1,550 nm) spectral region^7^.

An important aspect for future improvements of the technology or for new applications based on PCMs is a fundamental understanding and a thorough determination of their properties. The epitaxial growth of PCMs has established a new level of material quality^8–10^ and thus allows for an improved determination of their properties using techniques that normally cannot be applied to polycrystalline samples. For example, the electronic band structure of PCMs has been studied by angle-resolved photoemission spectroscopy^11,12^, which is not possible using polycrystalline samples. Furthermore, the ordering of vacancies has been studied in epitaxial GST alloys improving our understanding of the crystalline phases of PCMs^13^. Finally, the atomic stacking order of GeTe/Sb_2_Te_3_ superlattices has also been determined^14,15^.

The new insights offered by studying epitaxial PCMs clearly show the benefits of this approach. We therefore extend this approach by studying the optical and electrical properties of epitaxial Ge_2_Sb_2_Te_5_ (GST225) and compare them with the properties of α-GeTe and Sb_2_Te_3_, the binary compounds at the base of GST225 alloys. Even though these properties are well studied in polycrystalline GST225, we obtain new information on the electrical and optical properties of GST225. Specifically, the presence of an impurity band inside the energy gap of GST is observed.

### Experimental

The epitaxial α-GeTe, Sb_2_Te_3_, and GST225 films were grown by molecular beam epitaxy (MBE) on semi-insulating Si(111) substrates (*R* > 5,000 Ω cm) in order to allow for the electrical measurements, with double sided polishing for optical measurements. Standard substrate cleaning procedures were used, and Si(111)-√3 × √3-Sb surface reconstruction was created before the deposition. The deposition conditions that were applied are described in detail elsewhere^16,17^. After deposition, the samples were characterized by X-ray diffraction (XRD) using a PANalytical X′Pert Pro and Cu_Kα1_ radiation (λ = 1.540598 Å). The electrical characterization was performed using the van der Pauw geometry and a home-built Hall setup. The transmittance and reflectance spectra were recorded at room temperature using a Bruker IFS 66 v Fourier transform infrared (FTIR) spectrometer, which is evacuated during the measurements. The spectral resolution is set to 4 cm^−1^ or equivalently to 0.5 meV. The spectra in the range of 400−7,000 cm^−1^ (0.05−0.87 eV) were obtained using a deuterated triglycine sulfate (DTGS) detector and a Ge/KBr beam splitter, while in the range of 5,100–10,000 cm^−1^ (0.63−1.24 eV) we used a Ge diode detector and a Si/CaF_2_ beam splitter. In addition, the optical properties in the spectral range from 1.24 eV to 5.45 eV were investigated by means of spectroscopic ellipsometry using a Sopra GES5E ellipsometer and an angle of incidence of 75°. The ellipsometry data served as an additional reference for the fitting of the FTIR data, which is the main focus of the current paper. The transmittance, reflectance, and ellipsometry data were simultaneously fitted using the RefFIT program and by employing a two-layer (thin film + substrate) model. For the fits, the thickness of the layer as determined by XRD was used. Initially, the data of the film was fitted using seven to eight Drude-Lorentz functions. This resulted in a reasonable, but not perfect fit to the data. Finally, the fit to the FTIR data was improved using a variational dielectric function^18^.

### Structural characterization

**Figure 1:**
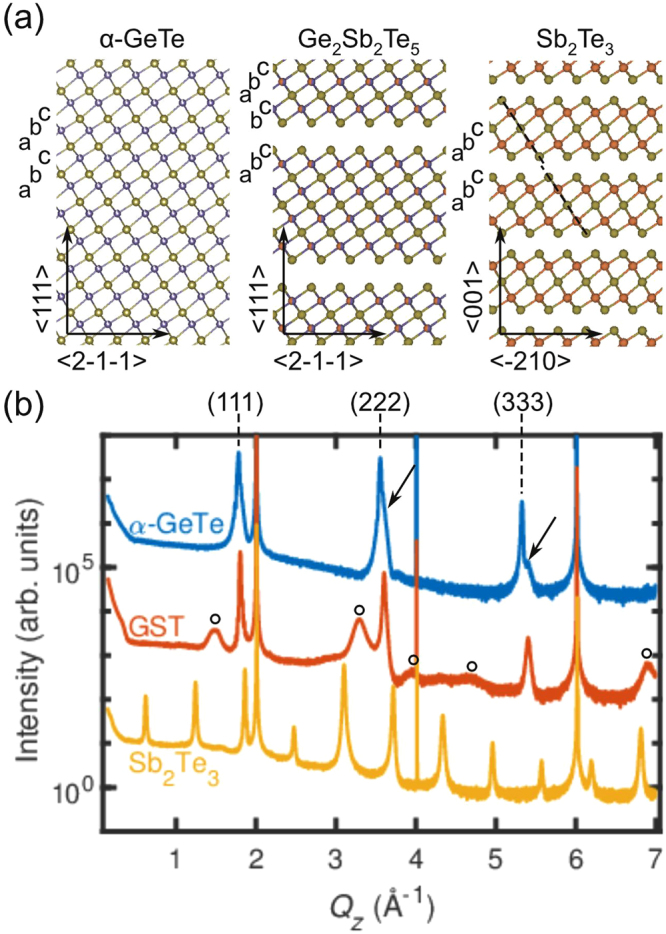
Structural characterization of epitaxial phase change materials. (**a**) Schematics of the crystal structures of α-GeTe, GST225, and Sb_2_Te_3_. The green, blue, and red circles represent Te, Ge, and Sb atoms, respectively. The arrows indicate the crystallographic directions and the letters a, b, and c the stacking sequence. The dashed lines in the right panel mark the horizontal shift of the quintuple layers in Sb_2_Te_3_. (**b**) XRD profiles of α-GeTe, GST225, and Sb_2_Te_3_ thin films plotted as a function of the reciprocal scattering vector *Q*_*z*_. The data are vertically shifted for clarity. The sharp peaks at 2, 4 and 6 Å^−1^ correspond to the Si(111), Si(222) and Si(333) diffraction peaks, respectively. The arrows point towards the shoulders on the right side of the α-GeTe (222) and (333) diffraction peaks and the circles indicate the broad features in the GST225 diffraction pattern. The XRD data show an obvious difference in crystal structure between the three materials.

In the schematics in Fig. 1(a), we show the crystal structures of the three investigated materials, while Fig. 1(b) displays typical XRD profiles of the epitaxial films of the three investigated phases. The sharp peaks at 2, 4 and 6 Å^−1^ correspond to the Si(111), Si(222) and Si(333) diffraction peaks, respectively. α-GeTe has a distorted rocksalt structure. For α-GeTe three high-intensity peaks are observed in the XRD profile that correspond to the (111), (222), and (333) diffraction peaks of α-GeTe. The shoulders on the right-hand side of these peaks, for example indicated by the arrows in Fig. 1(b), indicate that in some domains of the α-GeTe film the rhombohedral distortion is along one of the other equivalent 〈$\bar{1}11$〉 directions^16^. The stable composition of α-GeTe is Ge_0.85_Te^19^. There are thus a large number of vacancies (approximately 15%) at random positions on the Ge sublattice of α-GeTe.

GST225 has a rock salt structure in its metastable form with Ge and Sb on one sublattice and Te on the other. However, the XRD profile of the GST225 phase is distinctly different from the α-GeTe diffraction profile, because additional broad features are observed on the left- and right-hand sides of the main diffraction peaks (marked by the open circles). The peaks are due to an additional periodicity in the crystal, caused by the ordering of vacancies in vacancy layers^13^. Note that the chemical composition of Ge_2_Sb_2_Te_5_ can be considered as (Ge_0.4_Sb_0.4_)Te. Ge_2_Sb_2_Te_5_ thus contains 20% of vacancies on the Ge/Sb sublattice, which is approximately 30% more than α-GeTe. It turns out to be energetically favorable for the vacancies in GST225 to order into layers^20^, which can be observed by means of XRD. The fact that these peaks are broad can be related to the presence of disorder in the separation between the vacancy layers in the GST225 film^13^. From the peak positions, an average spacing between the vacancy layers of 2 nm is calculated, indicating that the composition of the GST225 film lies between the GST326 and GST225 phase^13^. Moreover, it is experimentally found that vacancy ordered GST retains the cubic rock salt stacking, although some regions with trigonal stacking are also present^13^. There is thus still a large degree of disorder present in the GST225 lattice, and the schematic in Fig. 1(a) only shows the ideal vacancy-ordered phase of GST225. Furthermore, note that the vacancy layers are present in addition to randomly distributed point defects that even exist in trigonal GST225^21^.

Sb_2_Te_3_ consists of quintuple layers separated by a van der Waals (vdW) gap. The vdW gap in Sb_2_Te_3_ (and in trigonal GST225 as well) can in principle be considered as a collapsed vacancy layer combined with a horizontal shift of the layers. As a consequence, the stacking order is different in this phase, and the atomic columns are shifted with respect to each other, as illustrated by the dashed line in Fig. 1(a). It is evident from the XRD profile in Fig. 1(b) that Sb_2_Te_3_ films exhibit an ordering: the number of observed peaks is three times as high as for α-GeTe. Compared with GST225, the peaks are sharp, indicating that there is more order in the out-of-plane direction. This is consistent with the Sb_2_Te_3_ crystal structure, because the separation between the vdW gaps in Sb_2_Te_3_ is constant throughout the film, which results in sharp diffraction peaks. The XRD measurements thus clearly show that the GST225 film is the only film of the three investigated phases with a significantly disordered crystal lattice, which is due to the clustering of vacancies. We would like to emphasize that this degree of disorder in GST225 is present in addition to existence of random point defects.

### Electrical characterization

**Figure 2:**
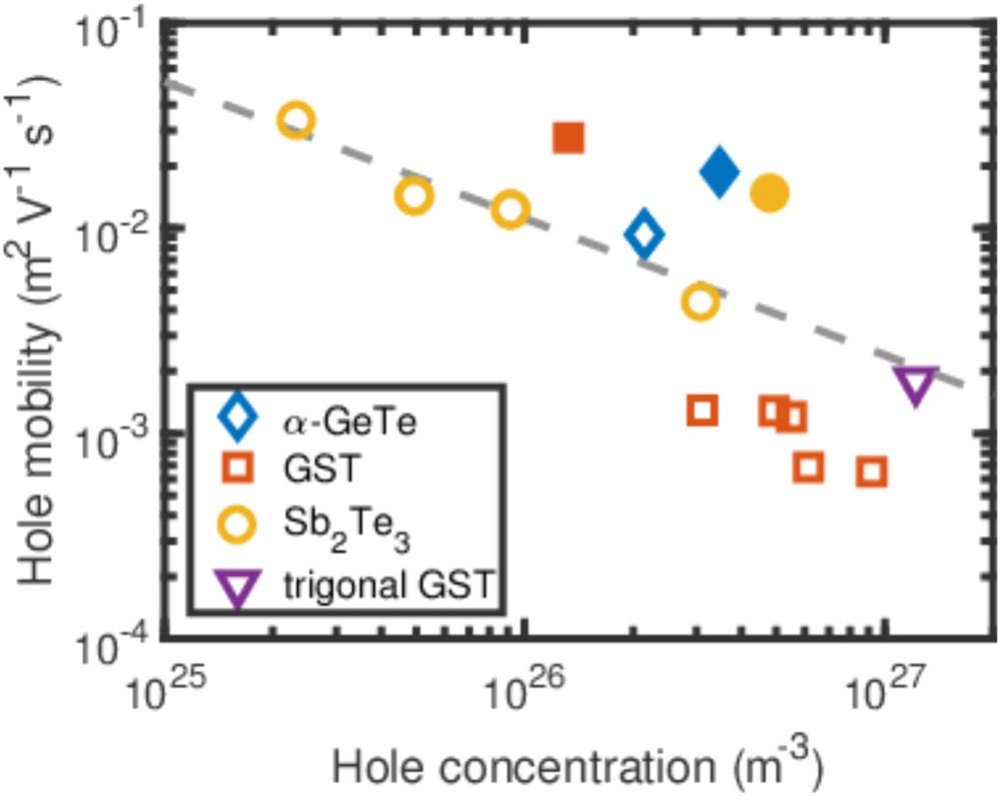
Electrical characterization at room temperature. Hole mobilities of α-GeTe, GST225, Sb_2_Te_3_, and trigonal GST225 as a function of the hole concentration. The data indicated by open symbols are determined by Hall measurements, whereas the data marked by full symbols are obtained from the optical measurements.

Hall measurements were performed at room temperature in order to determine the hole concentrations *p* and mobilities of the different materials. The open symbols in Fig. 2 show the room temperature mobilities of a number of Sb_2_Te_3_, GST225, and α-GeTe samples as a function of hole density. The mobility of Sb_2_Te_3_ strongly depends on the hole density. In fact, the mobility is proportional *p*^−2/3^ as shown by the dashed line. This trend was also observed for Sb-Sb_2_Te_3_ alloys measured at 0.3 K and indicates that the mean free path is determined by the distance between the dopants^22^. Interestingly, the mobility of α-GeTe and trigonal GST225 follows the same line. However, the mobility of the vacancy-ordered GST225 is significantly lower, but exhibits a similar trend. Siegrist *et al*.^23^ showed that the mobility of GST225 is strongly influenced by the presence of disorder. The source of disorder in cubically ordered GST225 arises, as already mentioned, not only from the slightly different spacing between the vacancy layers, but also from the presence of some atoms within the vacancy layers, which are not fully depleted as previously observed by scanning transmission electron microscopy (STEM)^13^. Based on the presence of such structural disorder, we conclude that the mobility in vacancy-ordered GST225 is limited by structural disorder due to the imperfect formation of vacancy layers. Note that a reduced mobility was also observed for Sb-Te alloys with a significant degree of disorder in good agreement with our conclusion^22^. This shows that, even though the disorder in the epitaxial GST225 film is sufficiently low to be on the metallic side of the Anderson metal insulator transition, the metallic conductivity is still influenced by the presence of disorder.

**Figure 3:**
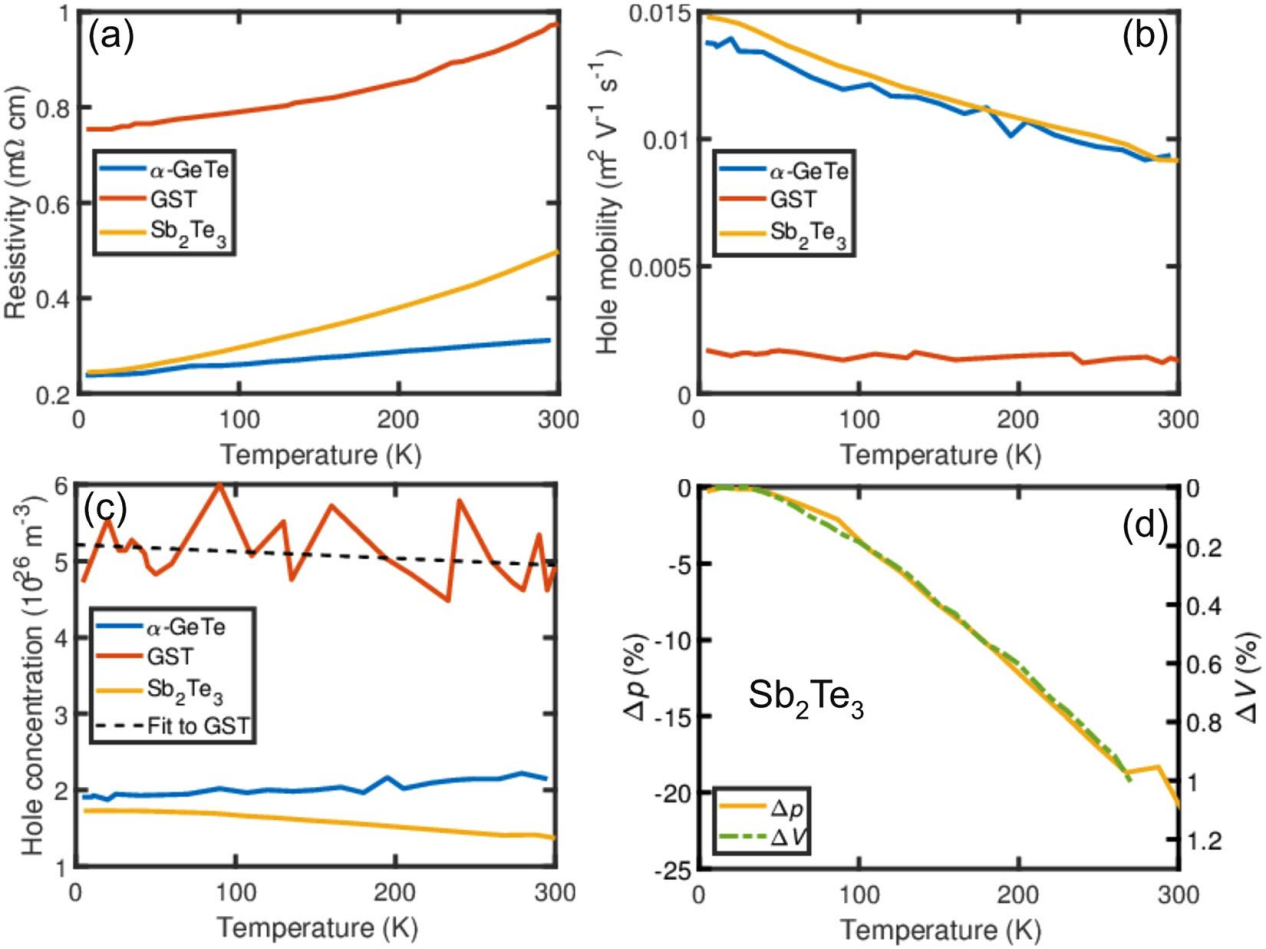
Temperature dependent electrical characterization. Temperature dependence of the (**a**) resistivities, (**b**) hole mobilities, and (**c**) hole concentrations for α-GeTe, GST225, and Sb_2_Te_3_ thin films. The non-linear increase of the resistivities of GST225 and Sb_2_Te_3_ is related to a decrease of the corresponding hole concentrations with increasing temperature. (**d**) Reduction of the hole concentration Δ*p* of Sb_2_Te_3_ compared with the expansion of the crystal lattice Δ*V*.

The temperature dependences of the resistivities of Sb_2_Te_3_, α-GeTe, and GST225 were measured and are shown in Fig. 3(a). A subtle difference can be observed in the temperature dependences of the resistivities. α-GeTe exhibits a linear increase of the resistivity with temperature, whereas the resistivity of Sb_2_Te_3_ and GST225 increases nonlinearly. A linear increase of the resistivity is typical for metals and due to the increased scattering with phonons with increasing temperature. For α-GeTe, we find that the temperature coefficients of the resistivity, here defined as the slope in the Fig. 3(a), has a value of 0.26 µΩ K^−1^. Temperature-dependent Hall measurements were performed in order to investigate the differences in the electric resistivities in more detail. We found that the hole mobilities of α-GeTe and Sb_2_Te_3_ decrease approximately linearly with increasing temperature as shown in Fig. 3(b), which is consistent with an increased phonon scattering rate. The temperature dependence of the mobility thus does not offer an explanation for the observed differences between the temperature dependences of the resistivities. However, the hole concentrations of these two samples exhibit a different behavior as shown in Fig. 3(c). For α-GeTe, the hole concentration increases slightly with increasing temperature. This is in good agreement with previous results^24^ and can be attributed to a decrease of the energy gap. For Sb_2_Te_3_, a decrease of *p* is observed. Such a decrease of *p* is typical for Sb_2_Te_3_, see for example Zhou *et al*.^25^, but is not well understood. Recent density functional theory (DFT) calculations show that the energy gap of Sb_2_Te_3_ increases with increasing temperature due to the thermal expansion of Sb_2_Te_3_^26^. This suggests that the increased energy gap is a possible cause of the reduced hole concentration. In order to verify if this is indeed the origin of the decrease of *p* in Sb_2_Te_3_, the change in the hole concentration Δ*p* and the change in unit cell volume Δ*V* evaluated from Chen *et al*.^27^ are compared with each other in Fig. 3(d). The good agreement between these two data sets is consistent with the assumption mentioned above. Unfortunately, the data for the GST225 samples turned out to be very noisy. A possible origin for the noise is the observed compositional disorder in the GST225 sample, i.e. the simultaneous presence of GeSb_2_Te_4_, Ge_2_Sb_2_Te_5_, and Ge_3_Sb_2_Te_6_ in addition to the simultaneous presence of vacancy layers and vdW gaps, whereas the other two samples exhibit pure phases. In addition, composition variations across the sample might be a challenge for the measurement. Nevertheless, the fit to the GST225 data (dashed line in Fig. 3 (c)) reveals a reduction of the hole concentration with temperature. This is consistent with the similar temperature dependence of the resistivities of Sb_2_Te_3_ and GST225. Furthermore, a reduction of the hole concentration with increasing temperature was also found for the stable trigonal phase of GST225^28^, indicating that it occurs in the stable and metastable phases of GST225.

### Optical characterization

**Figure 4:**
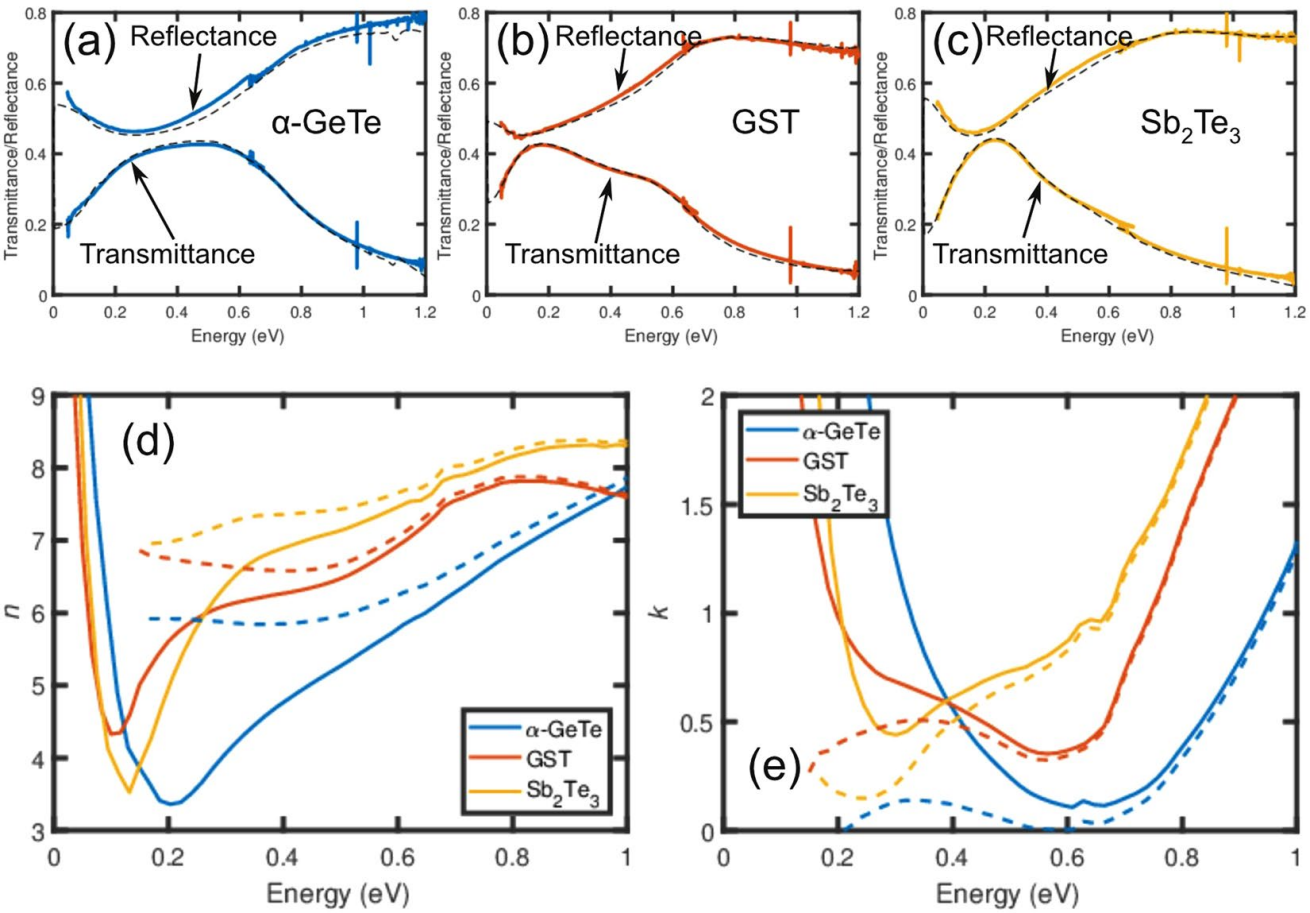
Optical characterization of epitaxial phase change materials. Transmittance (lower curves) and reflectance (upper curves) spectra of (**a**) α-GeTe, (**b**) GST225, and (**c**) Sb_2_Te_3_ thin films. The dashed lines indicate fits to the transmittance and reflectance data. (**d**) Real part *n* and (**e**) imaginary part *k* of the complex refractive index of α-GeTe, GST225, and Sb_2_Te_3_ calculated from the spectra in (**a**), (**b**), and (**c**). The solid lines represent the values of *n* and *k* optical constant including free-carrier absorption, whereas the dashed lines indicate the corresponding values without free-carrier absorption.

In this section, the optical properties of epitaxial PCMs in the spectral region up to 1 eV will be discussed. Figure 4(a–c) show the measured transmittances and reflectances of α-GeTe, GST225, and Sb_2_Te_3_ thin films, respectively, with a thickness of 36−38 nm. The transmittance (reflectance) data of the three samples show a similar characteristic. Starting from low energies, the transmittance (reflectance) increases (decreases) up to approximately 0.2 eV. The strong absorption at low energies is due to free-electron absorption within the same band. At higher energies, interband absorption occurs, and the transmittance (reflectance) decreases (increases). The transmittance and reflectance data were fitted as described in the experimental section in order to obtain the wavelength dependence of the real *n* and imaginary part *k* of the complex refractive index. The fits to the data are indicated by the dashed lines in Fig. 4(a–c) and the real *n* and imaginary part *k* of the complex refractive index are shown in Fig. 4(d,e), respectively. Based on the good fit of the model to the transmittance and reflectance data, we conclude that the model gives a good description of the complex refractive index of the film. In addition, the complex refractive indexes are calculated without the contribution of the free-carrier absorption (Drude peak). These are presented as the dashed lines in Fig. 4(d,e). Furthermore, some characteristic optical parameters such as the plasma frequency ω_*p*_, scattering time τ, and dc conductivity σ are also obtained from the fits and summarized in Table 1. From these quantities, the hole concentrations and mobilities of the three materials are calculated assuming effective hole masses of 0.3^23^, 0.35^12^, and 0.78^29^ for α-GeTe, GST225, and Sb_2_Te_3_, respectively. The calculated values are included in Fig. 2 indicated by full symbols. In general, the mobilities determined by FTIR spectroscopy are larger than the mobilities determined by the Hall measurements. This is especially true for the GST225 sample. Note that this observation is in agreement with previous investigations^23^, but that the origin of this difference remains unclear. A possible origin of this difference lies in the underlying assumptions made in the data analysis. The present data analysis of the electrical and optical data assumes that the conduction takes place in a single band. In fact the conduction in for example GST225 takes place in multiple bands with different effective masses^12^. Another possible reason for this difference is that the scattering cross-section of the point defects and the disorder are frequency dependent and more pronounced for dc transport (the electrical measurements) than for ac transport, probed by FTIR. Unfortunately, it is not possible to determine the exact origin of this difference from the current data set. Next, the optical gaps are determined from *k* after subtraction of the contribution of the free charge carriers as indicated by the dashed lines in Fig. 4(e). The optical gaps of Sb_2_Te_3_, GST225 and α-GeTe can be easily recognized and are determined by the energy where *k* (after subtraction of the Drude contribution) exhibits a minimum, i.e. 0.24 eV, 0.56 eV and 0.59 eV, respectively. The value for Sb_2_Te_3_ is in good agreement with the energy gap determined by scanning tunneling microscopy (STM)^30^, whereas the optical gap of α-GeTe is in a good agreement with results of DFT calculations^31^. For GST225, the optical gap is also in good agreement with recent STM data^32^. Lately, GeTe/Sb_2_Te_3_ superlattices were studied using FTIR^33^. It thus makes sense to compare those results with the results presented here. For the MBE-grown superlattices, an optical gap of 0.3 eV was found, which is in good agreement with our determination of the optical gap of Sb_2_Te_3_, indicating that the optical interband absorption in a GeTe/Sb_2_Te_3_ superlattice is dominated by Sb_2_Te_3_.

**Table 1:** Optical parameters determined by FTIR spectroscopy. ω_*p*_ denotes the plasma frequency, τ the scattering time, σ the dc conductivity, *E*_*g*_ the optical gap, and ε_**r**_ the corresponding values of *n* at zero frequency.

	α-GeTe	GST	Sb_2_Te_3_
ω_p_ (cm^−1^)	11,652	6,961	8,651
τ (fs)	33	42	64
σ (Ω^−1^ cm^−1^)	1.08 × 10^4^	4.808 × 10^3^	1.134 × 10^4^
E_g_ (eV)	0.59	0.56	0.24
ε_r_ (ω = 0)	35	42	48

Interestingly, the imaginary part of the complex refractive index *k* without the contribution of the Drude peak reveals additional features inside the energy gap of α-GeTe and GST225 with a maximum around 0.3 eV. For the latter material, this absorption feature is much stronger. Similar absorption features were found on other GeTe and GST225 samples and for GST225 also using surface sensitive techniques, such as two-photon ARPES12 and scanning tunneling microscopy30. Furthermore, it is important to point out that the FTIR characterization is a bulk sensitive characterization method. Therefore, it is concluded that the absorption feature is caused by the bulk properties of GeTe/GST225 and is not due to a measurement artefact. In the following a possible origin for the observed absorption feature is discussed. It is well known that Ge vacancies are the main point defect in α-GeTe^34^. However, single Ge vacancies do not create any states inside the energy gap^34^. In contrast, Te_Ge_ point defects do, but their formation energy is much larger than that of V_Ge_ point defects, and thus the formation is less likely to occur^34^. Recently, it was found that the clustering of vacancies can also result in electronic states in the center of the energy gap^20,35^. In view of the presence of a large amount of Ge vacancies in epitaxial α-GeTe films, this is indeed a possibility. Moreover, the clustering of vacancies is much more pronounced in ordered cubic GST225 as evident from the XRD investigations. Hence, more states inside the energy gap are expected, in good agreement with our observations. Therefore, we conclude that the states inside the energy gap are due to clustering of vacancies. Note that such an impurity band might act as a trap for optically excited electrons. In recent investigations of our GST225 samples using optical pump and THz probe measurements, an additional relaxation pathway was indeed observed^36^. Moreover, the presence of states inside the energy gap might be related to the observation of persistent photoconductivity in GST225 alloys^37^.

In view of the large interest in the dielectric properties of PCMs^38^, the real part of the complex refractive index *n* is shown in Fig. 4(d) both, with and without the free-electron contribution. We find that the value of *n* for α-GeTe between 0.2 and 1.0 eV is smaller compared to the ones for Sb_2_Te_3_ and GST225. This is mainly due to the larger energy gap of α-GeTe. The values of *n* for Sb_2_Te_3_ and GST225 are comparable, even though GST225 has a larger energy gap than Sb_2_Te_3_ and a much lower absorption coefficient, which is related to the value of *k*, at 0.6 eV. However, the absorption into the vacancy band results in a larger absorption coefficient in GST225 for energies below 0.4 eV. These two effects thus compensate each other and result in similar values of *n* for GST225 and Sb_2_Te_3_ around 0.1 eV. For completeness, we list the corresponding values of *n* at zero frequency [ε_*r*_ (ω = 0)] in Table 1. As expected, they show a similar trend. This supports the conclusion that the absorption into the vacancy band contributes to the high values of ε_r_ in PCMs. The maximum value on n is an important parameter for optical application, because it determines the reflectivity of the material. It is observed that this value is close to or exceeding 8 around 0.8 eV (1550 nm). This results in a real part of the dielectric function εr that is close to or exceeds 60. These values are significantly higher than the values obtained from polycrystalline samples by for example Shportko *et al.* (maximum ε_r_ = 48)^38^ or Park *et al.* (maximum ε_r_ = 44)^39^. This can be attributed to the close to perfect out-of-plane alignment of epitaxial films or an improvement of the crystalline quality. This indicates that the functional properties of optical devices based on GST225 can be enhanced by using epitaxial or textured GST225, because these devices commonly rely polycrystalline GST225 with a lower real part of the refractive index.

## Conclusions

The observation that both the hole concentration and the hole mobility of α-GeTe and Sb_2_Te_3_ change with temperature suggests that this should also occur for GST225. This is important for the modelling of phase change random access memory cells, because the melting of GST225 is due to Joule heating and thus depends on the electrical resistance. Moreover, the finding that the hole concentration changes with the lattice volume is also relevant for applications, because the expansion of PCMs is restricted in typical devices. This can thus influence the hole concentration and hence the resistance. These two effects should be taken into account for an accurate modelling of phase change memory devices, especially when devices containing GeTe/Sb_2_Te_3_ superlattices^40^ are considered. Furthermore, the observation of states inside the energy gaps in α-GeTe and GST225 gives a logical explanation for the observation of persistent photoconductivity in GST225^37^ and the presence of slow recombination channels for photoexcited carriers^36^. The obtained results underline the fact that epitaxial PCMs are very usefull for investigating the fundamental properties of PCMs. Further improvements of the quality of epitaxial PCMs such as a reduction of the hole concentration might improve the insights into the fundamental properties of this fascinating material class.
